# The human intestinal microbiome at extreme ages of life. Dietary intervention as a way to counteract alterations

**DOI:** 10.3389/fgene.2014.00406

**Published:** 2014-11-21

**Authors:** Nuria Salazar, Silvia Arboleya, Lorena Valdés, Catherine Stanton, Paul Ross, Lorena Ruiz, Miguel Gueimonde, Clara G. de los Reyes-Gavilán

**Affiliations:** ^1^Department of Microbiology and Biochemistry of Dairy Products, Instituto de Productos Lácteos de Asturias – Consejo Superior de Investigaciones CientíficasVillaviciosa, Spain; ^2^Alimentary Pharmabiotic Centre, Teagasc, Food Research Centre MooreparkFermoy, Ireland; ^3^Alimentary Pharmabiotic Centre, University College CorkCork, Ireland

**Keywords:** intestinal microbiome, elderly, newborns, dietary intervention, probiotics

## Abstract

The intestinal microbiome is defined as the assembly of genomes from microorganisms inhabiting the gut. This microbial ecosystem regulates important functions of the host and its correct composition and functionality is essential for a “healthy status.” Metagenomic studies have highlighted variations of the intestinal microbiota as a function of age and diet. Colonization of the infant gut starts at birth and is influenced by feeding habits (formula vs. breast-feeding), birth mode and antibiotic exposure. The intestinal microbiota of full-term vaginally delivered breast-fed infants is considered the gold-standard, representing the reference for studies of alterations in other pediatric populations. At 2–3 years of age, the intestinal microbiota reaches a composition similar to adults, remaining without noticeable variations until senescence, when microbial instability and changes reappear. Here we summarize the current knowledge on intestinal microbiota alterations at extreme stages of life and tools for designing differentiated nutritional strategies by the use of probiotics, prebiotics and specific nutrients in order to restore a balanced microbiota and to improve immune and nutritional status.

## INTRODUCTION

It is increasingly clear that the gut microbiota is essential for maintaining health. Until recently its role in human health had remained largely unknown, mainly due to the lack of methods to study unculturable microorganisms, which constitute a large fraction of the gut microbiota. However, in recent years the development of next generation sequencing (NGS) methods has facilitated the performance of metagenomic studies to determine gut microbiome composition. These methods, together with the use of gnotobiotic animals have demonstrated the importance of the microbiota for host health, providing the basis for the use of dietary interventions aimed at counteracting microbiota aberrancies.

Next generation sequencing have indicated that, in spite of a high inter-individual variability, human intestinal bacterial populations of adults can be classified into three robust clusters called “enterotypes,” dominated by *Bacteroides*, *Prevotella*, and *Ruminococcus* (Arumugam et al., 2011). These enterotypes seem independent of nationality, age, gender, and body mass index but are influenced by long-term dietary habits (Wu et al., 2011). In addition, some microbiota alterations are associated with different human diseases (Turnbaugh et al., 2009; Qin et al., 2010; Karlsson et al., 2013). However, to date most of the larger studies have focused on adult populations and we are still far from clearly understanding as to how the microbiota affects health at different life stages. Interestingly, whilst the microbiota shows a high stability and resilience to change during adult life, it seems to be far more unstable at early infancy and senescence.

The initial steps of microbiota establishment in the newborn are critical for a proper development (El Aidy et al., 2013; Borre et al., 2014), and may be affected by several factors (Johnson and Versalovic, 2012). During the initial colonization the microbiota suffers microbial succession phenomena and is unstable (Yatsunenko et al., 2012). Similarly, at senescence the microbiota suffers new changes, becoming unstable again (Claesson et al., 2011, 2012; Salazar et al., 2013). Therefore, it is at these extremes of life where strategies aiming at microbiome modulation may have a deeper impact on health.

## CULTURE INDEPENDENT AND OMIC TECHNIQUES FOR THE STUDY OF THE INTESTINAL MICROBIOTA

Most studies on the intestinal microbiota have focused on microbial communities and their variation among human subgroups, in order to define which microorganisms are in the gut and how their presence/absence correlates with human health. Compositional variation can be detected through polymerase chain reaction-denaturing gradient gel electrophoresis (PCR-DGGE) of 16S rRNA gene, coupled to Sanger sequencing (Huipeng et al., 2014). Fluorescence *in situ* hybridization (FISH) and quantitative PCR (qPCR) also allow quantification of selected bacteria in mixed populations, but requires design of probes/primers and therefore cannot detect unknown or low abundance microbial groups. Therefore, total 16S rRNA gene sequencing through NGS platforms (454-Pyrosequencing, MiSeq, Ion Torrent, or SOLID) is becoming the gold-standard for overcoming the limitations of the aforementioned methods. NGS platforms are improving to offer cheaper alternatives and better resolution to detect unculturable, unknown and low frequent bacteria (Claesson et al., 2011; Fouhy et al., 2012; Mitra et al., 2013). Significant bioinformatic efforts are required to analyze the millions of reads generated through NGS. Alternative high-throughput methods include oligonucleotide microarrays (PhyloChip, Microbiota Array, HuGChip, and HITChip), that determine the presence and abundance of thousands of phylotypes in a sample (Paliy and Agans, 2012; Tottey et al., 2013) although *de novo* probe design, optimization and validation is time consuming (van den Bogert et al., 2011). Microarray and NGS showed good correlation though minor differences may appear depending, among other factors, on the origin of samples and number of 16S rRNA gene copies. Both techniques can be complementary: microarray technology is a powerful method for routine study of many samples whereas NGS enables a detailed understanding of microbial gene diversity (Roh et al., 2010).

Current efforts focus on shotgun metagenomic sequencing to generate comprehensive gene catalogs reflecting what metabolic functionalities the intestinal microbiota encodes (Qin et al., 2010; Cotillard et al., 2013; Rampelli et al., 2013). Despite the large compositional variation among healthy individuals, distribution of functionality is more conserved, suggesting that microbiota metabolic potential might be more relevant than its taxonomical composition. Metagenomics also revealed activities exclusive from gut environments and provided the basis to describe new vitamins synthesis and signaling pathways (Jones et al., 2008). Moreover, shotgun sequencing coupled with *in vitro* enrichment cultures from fecal samples, allowed complete genomes assembly of unculturable methanogenic archaeons, demonstrating that low frequency bacteria encode functionalities that might be relevant in the cross-talk between microbiota and host (Borrel et al., 2012; Luo et al., 2012). Functional microarrays are also available such as the CAZymes Chip that detects thousands of carbohydrate degrading activities encoded by a microbiota sample (El Kaoutari et al., 2013).

What gut microbes are actually doing can be investigated through a combination of metatranscriptomics, metaproteomics, and metabolomics approaches to analyze RNA, protein and small metabolite pools from feces (Gosalbes et al., 2011; Marcobal et al., 2013; Franzosa et al., 2014; Ursell et al., 2014), which are subsequently identified by comparison against appropriate databases. Comprehension of the role that human microbiota plays in health and disease requires integration of taxonomical and metabolic features as well as the understanding of the cross-talk between microbiota and host. Metabolomics provide the basis to evaluate dietary intervention’s effects on gut microbiota composition/functionality and host metabolic outcomes (Marcobal et al., 2013; Daniel et al., 2014). Development of computational tools to integrate results from multiple levels still remains a challenge.

## INTESTINAL MICROBIOTA, IMMUNE SYSTEM, AND NUTRITIONAL STATUS THROUGHOUT LIFE

### NEWBORNS

Traditionally, it was thought that microbial colonization of the gastrointestinal tract begins immediately after birth. Recent studies have shown the presence of microorganisms in placenta, amniotic fluid, umbilical cord blood, and meconium (Jiménez et al., 2005; DiGiulio, 2012; Moles et al., 2013; Aagaard et al., 2014). The classical pattern of early microbial development involves a first colonization by facultative anaerobes, which deplete the initial oxygen supplies creating a more suitable environment for the subsequent colonization by strict anaerobes (Fanaro et al., 2003). From an initial low diversity and complexity the intestinal microbiota reaches a stable population similar to that of an adult around 2–3 years of age (Koenig et al., 2011; Yatsunenko et al., 2012). The microbial population established in the initial stages of life has a profound impact on epigenetic programming and future homeostasis and well-being of the individual (Palmer et al., 2007; Canani et al., 2011). In this way, it is known that maternal and neonatal under-nutrition may promote an inadequate gut microbiota composition and functionality, accounting for deviant programming of later immunity and of regulation of genes involved in lipid and carbohydrate metabolism (Canani et al., 2011; Kerperien et al., 2012).

Several factors influence the establishment and composition of the infant intestinal microbiota: feeding type, delivery mode, gestational age or the use of antibiotics, among others (Penders et al., 2006). Breast milk is considered the optimal feeding pattern for newborns and the WHO recommends exclusive breast-feeding up to 6 months of age and then supplemental breast-feeding up to 2 years of age (WHO, 2009). Short- and long-term health benefits associated with breast-feeding have been reported and breast-fed infants are considered healthier, showing lower incidence of enteric and non-enteric infections, necrotizing enterocolitis (NEC), allergy and atopic disorders or diabetes compared with formula-fed infants (Le Huerou-Luron et al., 2010). Breast milk contains a wide range of protective compounds including carbohydrates (such as oligosaccharides with strong prebiotic activity and ability to displace pathogens), nucleotides, immunoglobulins, cytokines, short chain fatty acids (SCFA), and lactoferrin, and is a source of beneficial bacteria (Marques et al., 2010; Solís et al., 2010; Bode, 2012). The intestine of breast-fed infants was traditionally considered to be dominated by bifidobacteria but recent studies using high-throughput 16S rDNA techniques demonstrated that Proteobacteria dominate the infant intestinal microbiota (Arboleya et al., 2012a; Fan et al., 2014). However, breast-fed infants presented higher levels and abundance of bifidobacteria and lower levels of potential pathogens than formula-fed babies, in which a more diverse microbiota dominated by *Bacteroides* and Clostridia resides, with higher levels of *Klebsiella* (Fallani et al., 2010; Bezirtzoglou et al., 2011). Differential patterns of SCFA production have been found in breast-fed vs. formula-fed infants (Le Huerou-Luron et al., 2010; Arboleya et al., 2012b). Weaning promoted higher levels of Bacteroidetes and Firmicutes as well as functional genes characteristic of adult microbiome whereas it decreases Bifidobacteria and Proteobacteria (Fallani et al., 2011; Koenig et al., 2011). The establishment of the so-called human “enterotypes” seems to start around 18 months, although at this early age these enterotypes are still susceptible to shifting (Bergstrom et al., 2014).

### ADULTS

The human gut microbiota reaches its maximum complexity at adolescence following a gradual increase in the phylogenetic diversity of the microbiome during adulthood. The gut of healthy adults is populated by around 10^14^ bacteria which outnumber the total eukaryotic cells of the human body. This ecosystem is dominated by the phyla Bacteroidetes and Firmicutes while Actinobacteria, Proteobacteria, and Verrucomicrobia are represented in lower percentages (Arumugam et al., 2011). In addition to bacterial and eukaryotic cells, the main representative of *Archaea* in the adult gut is the methanogenic species *Methanobrevibacter smithii* (Eckburg et al., 2005).

The human microbiome contributes to shaping the immune system and should not be underestimated as a key factor in the maintenance of human health, being a key player in vitamin production, digestion, energy homeostasis, angiogenesis and maintenance of intestinal barrier integrity. The human gut microbiota appears altered in several disease states including obesity, inflammatory bowel disease and irritable bowel syndrome. The responses of the microbiota to antibiotic treatment can vary depending on individuals and is influenced by the prior exposure and the type of antibiotic used (Sullivan et al., 2001).

The “adult-like” gut microbiota ecosystem maintains a high stability and homeostasis in the absence of significant ecological stressors but intestinal dysbiosis appears with the old age.

### ELDERLY

Aging has been defined as “the regression of physiological function accompanied by advancement of age” (Woodmansey, 2007) and is associated with physiological changes in the gastrointestinal tract, as well as changes in dietary patterns and immune function. Aging is intimately linked with a decline in the normal function of the immune system (Haq and McElhaney, 2014) which results in an increased vulnerability of individuals against common infections (immunosenescence). Although physiological phenomena linked to aging vary among people and is influenced by external factors, according to the WHO most westernized countries have accepted the chronological age of 65 years as a definition of “elderly.” Malnutrition is a classical feature of old age and is linked to physiological changes that impact food digestion and absorption. Increased threshold for taste and smell, swallowing difficulties and masticatory dysfunction in the elderly can result in nutritionally imbalanced diets. Elderly people also suffer from atrophic gastritis with a decreased absorption of calcium, ferric iron, and vitamin B12 (Biagi et al., 2012). In addition, reduced intestinal motility leads to fecal impaction and constipation and an increased intestinal transit time is linked with reduced bacterial excretion and augmentation of bacterial protein fermentation, which consequently alters the gut fermentative process (Biagi et al., 2012).

The imbalance between pro-inflammatory and anti-inflammatory status in aged people results in a low-grade chronic systemic inflammation known as “inflammaging.” Inflammaging contributes to frailty, which is considered a hallmark of unhealthy aging, and degenerative disorders (Biagi et al., 2012). The main changes in the immune system include reduced humoral responses, decreases in dendritic cells efficiency to activate T and B cell populations, declination in the generation of new naive T and B cells, and reduced natural killer cell activity (Vallejo, 2011). Also augmented levels of pro-inflammatory cytokines such as TNF-α, IL12 and the chemokine IL8, and a reduction in NK cells cytotoxic activity have been reported (Salazar et al., 2013). These alterations contribute to the prevalence of cancer, autoimmune and chronic diseases such as Alzheimer’s disease, atherosclerosis, osteoarthritis and insulin resistance (Haq and McElhaney, 2014). The origins and drivers of the age-related chronic inflammation are not entirely clear but there is a growing body of evidence suggesting the role of the age-related intestinal microbiota in inflammaging.

The core microbiota composition of elderly individuals significantly differs from healthy adults and the set of microbial genes required for the proper maintenance of the gut homeostasis is likely to be different than that required for younger populations (Qin et al., 2010). The abundance of the members of the dominant phyla of the gut microbiota, Firmicutes and Bacteroidetes in elders is controversial and seems to be country dependent (Biagi et al., 2012). A higher proportion of Bacteroidetes and a reduced stability of the microbial community has been reported in the elderly Irish population, whereas the proportion of Bacteroidetes remains constant in Italian elderly (Biagi et al., 2013). The age-related intestinal microbiota changes include a reduction in species diversity of most bacterial groups which makes microbiota less resistant to major fluctuations in response to several environmental factors. Shifts in the dominant species within several bacterial groups, decline in beneficial microorganisms, increase of facultative anaerobic bacteria and decrease in the availability of total SCFA have also been described (Salazar et al., 2013). These modifications of the intestinal microbiota may contribute to the establishment of a state prone to disease and might increase the susceptibility to infections, such as those caused by *Clostridium difficile* (Biagi et al., 2012).

## NUTRITIONAL STRATEGIES FOR RESTORING A BALANCED MICROBIOTA AT EXTREME AGES OF LIFE

The microbiota of specific healthy human groups is considered a reference to identify targets for intervention in order to promote a balanced microbiota in populations at extreme ages of life. The gold standard microbiota in infants is that of full-term vaginally delivered breast-fed babies. Early stages of intestinal colonization when the microbiota is still developing, is the key period to apply modulation strategies. Identified alterations of the infant microbiota generally impact on microbial diversity, anaerobe/facultative anaerobes ratio, incidence and levels of *Enterobacteriaceae* and intestinal pathogens, SCFA production pattern, and functionality of metagenomes (Arboleya et al., 2012a,b,d). Lowering the incidence and severity of NEC as well as the infection risk in premature infants is a target of relevance (Wang et al., 2009). The main targets for nutritional intervention in order to restore a balanced microbiota in newborns are summarized in **Table 1**.

**Table 1:** Main targets for nutritional intervention in order to restore a balanced microbiota at extreme ages of life.

Human groups	Intestinal microbiota alterations identified	Impact on extra-intestinal locations	Experimental techniques for studying the intestinal microbiota	Reference
***Newborns***
**Formula-fed**	✓ Dominated by *Bacteroides* and Clostridia ✓ Increased *Atopobium* levels ✓ Higher levels of potential pathogens, such as *Clostridium difficile* ✓ Elevated propionate levels ✓ Higher diversity	✓ Risk of obesity in childhood and adulthood ✓ Induction of Th1 cytokines in children ✓ Worse neurodevelopmental outcome than breast-fed infants	FISH 16S rRNA gene sequencing GC	Fallani et al. (2010), Bezirtzoglou et al. (2011), Azad et al. (2013), Borre et al. (2014), Imai et al. (2014), Winkler et al. (2014)
**Premature**	✓ Lower levels of *Bifidobacterium* and *Bacteroides* ✓ Elevated levels of potential pathogens, such as *Klebsiella pneumoniae* ✓ Elevated facultative anaerobe to strict anaerobes ratio ✓ Reduced levels of SCFA ✓ Lower diversity	✓ Immature system immune ✓ Lower cognitive capacities ✓ Risk of infections, NEC and death	qPCR PCR-DGGE/TGGE 16S rRNA gene sequencing GC	Begega et al. (2010), Jacquot et al. (2011), Arboleya et al. (2012b), Barrett et al. (2013)
**C-section delivery**	✓ Dominated by skin microorganisms: *Staphylococcus*, *Corynebacterium*, Propionibacterium ✓ Elevated levels of *Escherichia, Shigella, Bacteroides* ✓ Reduced levels of *Bifidobacterium* ✓ Lower diversity	✓ Risk of asthma, allergies or atopic diseases in adulthood ✓ Risk of obesity in adulthood ✓ Risk of diabetes I	qPCR PCR-DGGE/TGGE 16S rRNA gene sequencing	Salminen et al. (2004), Biasucci et al. (2010), Dominguez-Bello et al. (2010), Goldani et al. (2011), Azad et al. (2013), Borre et al. (2014)
**Antibiotic treatment**	✓ Elevated levels of enterobacteria and enterococci ✓ Lower levels of *Bifidobacterium* ✓ Lower diversity	✓ Risk of asthma, allergies in adulthood ✓ Risk of obesity in adulthood	qPCR FISH 16S rRNA gene sequencing	Tanaka et al. (2009), Fallani et al. (2010), Fouhy et al. (2012)
***Elderly***
**Healthy elderly**	✓ Country dependent changes in Firmicutes/Bacteroidetes ratio ✓ Elevated facultative anaerobes ✓ Reduced level SCFA ✓ Lower diversity	✓ Decline in the normal function of the immune system ✓ Risk of autoimmune and chronic diseases ✓ Poor response to vaccination ✓ Increased vulnerability to infection	qPCR 16S rRNA gene sequencing GC	Claesson et al. (2011), Biagi et al. (2013), Salazar et al. (2013), Haq and McElhaney (2014)
**Elderly under medical treatment**	✓ Lower levels Bacteroidetes, *Bifidobacterium, Clostridium* cluster XIVa, *Faecalibacterium prausnitzii* members (Cluster IV) ✓ Increased levels of *Lactobacillus* after antibiotic therapy ✓ Lower diversity	✓ Increased susceptibility to infections by *C. difficile*	Culture-based techniques qPCR 16S rRNA gene sequencing	O’Sullivan et al. (2012), Biagi et al. (2013), Keller and Surawicz (2014)

In the elderly, the microbiota of a younger population of healthy adults otherwise sharing similar characteristics in terms of geographic location and diet as the older people is considered the benchmark of healthy microbiota. This reference group must have had a past socio-economic status as similar as possible to the elders: a history of exposure to different environments in the two populations may lead to attribute to aging, variations due to the action of different environmental factors such as those linked to social habits, nutritional patterns, or residence location (Claesson et al., 2012; Salazar et al., 2013). Loss of the community-associated microbiota, that is the set of microorganisms shared by individuals from a given social group, correlated with increased frailty. The reduced levels of *C. leptum* and *Blautia coccoides* and the higher incidence of *C. difficile* infections are relevant targets (Salazar et al., 2013; Power et al., 2014). Some nutritional deficiencies of elders are related with lower intakes of specific nutrients which are important for maintaining the immune and gastrointestinal functions and which are a consequence of the general loss of functionality of the gastrointestinal tract (González et al., 2013; Power et al., 2014). Therefore, nutritional strategies in elders should consider the intestinal microbiota, immune system, and nutritional deficiencies (**Table 1**).

Different approaches have been proposed for restoring a balanced microbiota. Fecal transplantation is known from many years ago (Eiseman et al., 1958); although a renewed interest has arisen in recent time, this alternative has currently important ethical concerns and legal barriers (Ratner, 2014); however, it could be considered as proof of principle research that would help to find a definite cocktail of strains to be administered as a corrective measure of intestinal dysbiosis (Petrof et al., 2013).

**FIGURE 1:**
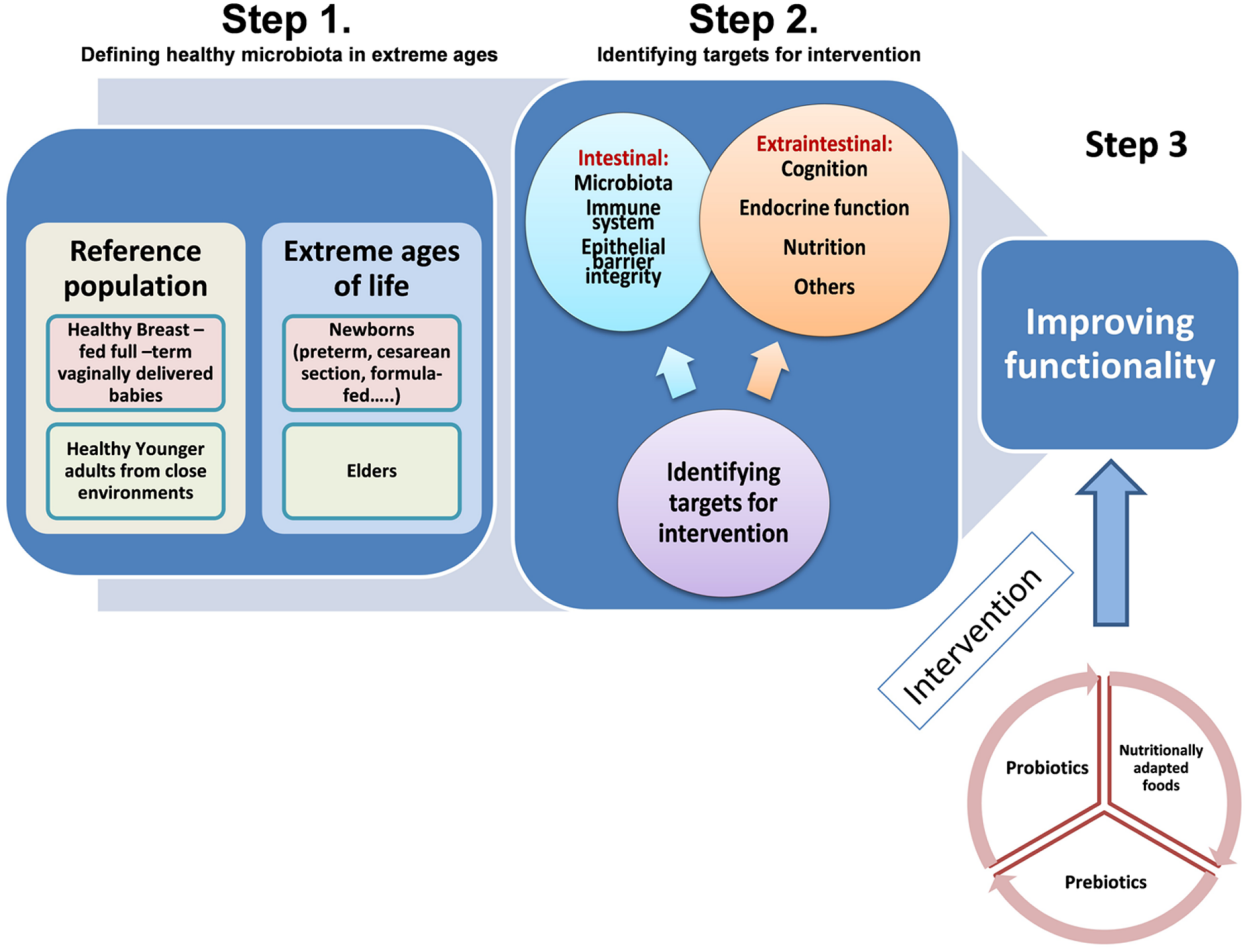
**Schematic representation of the steps involved in designing nutritional interventions to improve functionality of specific human population groups in the extreme ages of life: (newborns and elders).** Step 1: defining reference populations for comparative studies. Step 2: identifying intestinal and extraintestinal targets for intervention. Step 3: proving functional efficacy of different nutritional strategies.

Probiotics are “live microorganisms which when administered in adequate amount confer a health benefit on the host” (FAO/WHO, 2006). The most used probiotics in foods are lactobacilli and bifidobacteria. Probiotics may interact with the intestinal microbiota, inhibit enteric pathogens and modulate the immune system. Prebiotics are “selectively fermented ingredients that result in specific changes in the composition and/or activity of the gastrointestinal microbiota, thus conferring benefit(s) upon host health” (Roberfroid et al., 2010). Prebiotics usually are complex carbohydrates naturally present in vegetables and other foods or can be industrially produced as ingredients. Their beneficial effects are linked to promotion of changes on the composition and metabolic activity of the intestinal microbiota (Roberfroid et al., 2010). The inclusion of probiotics and prebiotics in foods has been extensively used for modulating the gut microbiota. However, to date the specific needs of defined target populations, such as the extremes of life, in terms of microbiota modulation have not been taken into account for probiotic or prebiotic selection and products formulation. A schematic representation of the steps involved in designing nutritional intervention strategies for human extreme ages is provided in **Figure 1**.

## INTEGRATED PERSPECTIVE AND FUTURE TRENDS OF THE HUMAN INTESTINAL MICROBIOTA AND DIET

The development of dietary intervention strategies specifically targeted to populations at the extreme ages of life, may constitute an interesting approach (Arboleya et al., 2012c). A number of studies support the benefits of specific probiotics and prebiotics in adults (Power et al., 2014), although the evidence is still scarce in infants and elders. Several studies indicate that prebiotics may be effective in decreasing the rate of overall infections in infants (Lohner et al., 2014) and that maternal probiotic supplementation may decrease the incidence of NEC in breastfed preterm babies (Benor et al., 2014). However, there is still a need for long-term follow-up of initiated probiotic studies to assess the impact of early life interventions for late efficacy (Videhult et al., 2014). Therefore, the selection of probiotics and prebiotics included in tailor-made foods for human populations at extreme ages of life and targeting well defined microbiota alterations, is a key future action (Aggett et al., 2003; Adlerberth and Wold, 2009; Duncan and Flint, 2013). This is pointed out by recent reports underlining a high inter-individual variability in the response to probiotics (van Baarlen et al., 2011; Grzeskowiak et al., 2012) and important differences in the effect of the same probiotics and prebiotics on the intestinal microbiota from different human groups (Arboleya et al., 2013).

Moreover, considering the wide impact of the microbiota on host physiology it may be time for addressing new targets such as the age-related chronic-inflammation (Macpherson and Harris, 2004) or the maintenance or correct development of cognitive function in elders and preterm babies, respectively (Gilbert et al., 2013). In addition, these population groups may require specific and nutritionally adequate products able to satisfy their particular requirements. Such knowledge should now be integrated into the development of a new generation of highly adapted functional food products.

## Conflict of Interest Statement

The authors declare that the research was conducted in the absence of any commercial or financial relationships that could be construed as a potential conflict of interest.
